# A List of the Diseases Admitted under the Care of the Physicians of the Universal Dispensary for Children, St. Andrew's Hill, Doctors' Commons; from the 1st of January, 1818, to the 1st of April Following

**Published:** 1818-05

**Authors:** J. B. Davis, Wm. Shearman

**Affiliations:** Physicians; Physicians


*A List of the Diseases admitted under the Care of the Physicians of the Universal Dispensary for Children, St. Andrew's Hill, Doctors' Commons; from the* 1*st of January,* 1818, *to the* 1*st of April following.*

| Diseases. | Cases. | Diseases. | Cases. |
|---|---|---|---|
| Febris | 6 | Hydrocephalus Acutus | 8 |
| —— Remittens | 5 | ———— Chron. | 7 |
| —— Catarrhalis | 10 | Pneumonia | 18 |
| Febricula | 10 | Pleurodynia | 2 |
| Tussis | 19 | Icterus | 4 |
| —— cum Febre | 7 | Erythema | 4 |
| —— cum Dyspnœa | 4 | Erysipelas | 5 |
| Pertussis | 53 | Hydrops | 4 |
| Urticaria | 4 | Dyspepsia | 13 |
| Roseola | 3 | Epistaxis | 1 |
| Eruptiones Variæ | 6 | Cynanche Trachealis | 14 |
| ———— cum Febre | 3 | Lumbrici | 11 |
| Diarrhœa | 35 | Ascarides | 9 |
| Dysenteria | 10 | Tænia | 2 |
| Cholera Morbus | 11 | Chorea Sancti Viti | 7 |
| Convulsiones | 15 | Hæmoptoe | 2 |
| Atrophia | 4 | Spasmi Musculorum Manuum et Pedum | 4 |
| Tabes Mesenteric a | 5 | Rheumatismus Acutus | 5 |
| Tormina | 6 | Rachitis | 12 |
| Vomitiones | 3 | | |
| Morbilli | 18 | | |
| Varicella | 1 | | 370 |

Although it appears, by the above return, that the number of children admitted as patients into the Universal Dispensary for Children has been considerable since Christmas, yet the general character of disease among them has been mild; at least, until the month of March. In January and February, Measles and Hooping Cough were prevalent, but far from severe. Scarcely a case ended fatally. Diarrhœa and Catarrh, singly, and in conjunction, formed a principal feature in the diseases of the season; but were mild and tractable. In the beginning of March, from fifteen to twenty cases of Croup were brought to the Dispensary. The symptoms were of the most urgent nature, and indicated a high degree of inflammation in the membrane of the larynx and trachea. In the course of thirty-six hours from the attack, this disease became combined with Pneumonia; the face quickly changed from a florid to a purple hue, the lips were dark and swollen, the eyes prominent, the breathing short and laborious and seemingly painful, the voice extremely hoarse, and expiration stridulous, accompanied with a hard irritative cough, the pulse too rapid to admit of being numbered, and pyrexia intense. In this state, two children expired, after having laboured under the disease five days. Three cases of well marked Erysipelas Infantile, in children under seven weeks old, were treated with success. One of them had an erysipelatous intumescence from head to foot, of a deep red colour, which, as the pyrexia subsided, grew pale, and disappeared in extensive desquamations. The eye-lids in this child were so swollen and tense for eight or ten days, as to completely close the eyes, from the inner canthi of which flowed copiously a purulent secretion. The eye-lids, face, and head, formed together a shapeless mass of swelling.

A Fever, partaking of a bilious character, was frequent in the month of March. Several cases of genuine acute Rheumatism were seen in children of three and four years of age. Catarrhal affections were also common in this month ; and not unfrequently ended in pneumonia. The character of Hooping Cough very severe.

Many children with dilated pupils, prominent eyes, and large heads, who had for months, at times, been attacked with convulsive motions of the whole body, have become patients of the Institution, labouring under a spastic rigidity of the lower and upper extremities. The arms and fingers of these patients remain in an extended state for hours and days together, and are in general so exceedingly stiff as not to be readily bent by the hand of another person ; and if flexion of their fingers be thus promoted, they resume, shortly after, their former state of rigidity, and continue in it. These children have slight pyrexia, and are dull and inactive. The upper and lower extremities are not, however, equally stiff at the same time. If the fingers and arms be rigid, the legs are frequently flexible. If these, on the contrary, be in a state of rigidity, the former are observed to regain their natural flexibility.

J. B. DAVIS,

WM. SHEARMAN, } Physicians.

PART

